# Restoring Teeth in Porcelain without Shoulder Preparation

**Published:** 1922-05

**Authors:** Albert Leland Legro


### RESTORING TEETH IN PORCELAIN WITHOUT SHOULDER PREPARATION

ALBERT LELAND LEGRO, D.D.S.

The essayist reported the results of an examination recently made in seventy-two cases of well-executed porcelain jacket restorations with shoulder preparation. These cases were from his own practice and two other practices. The crowns had been in position three and a half years or more. Sixty-four cases showed recession of the gum tissue. In twenty-two of these cases, the recessions apparently resulted from poor carving of the portion of the crown against the soft tissues. Thirty-two cases showed preparation of the roots straight around the tooth with no attempt to follow the line of the cemento-enamel junction. In eleven cases the pulps were dead, and in five of these the roots were dark. These cases showed that too much dentine had been removed and the shoulders had been made too wide, with inadequate protection for the pulps. Eight cases showed no apparent pathological conditions. Thermal response was active in forty-seven cases, though pain was manifested in only seven cases, where cold could not be tolerated. Thirtyseven cases responded very actively to electrical tests; twenty-four cases showed different degrees of sluggishness.

The entire number showed the more or less disintegration of the remaining enamel, and the percentage of broken-down enamel was appalling. In at least six cases one-third of the enamel shoulder was missing All the cases were in the mouths of patients of good physical resistance.

In response to an inquiry by Dr. Thompson during the discussion, Dr. LeGro replied that the number of cases was too small to be conclusive, but said that he had made investigation only recently for the purpose of bringing the facts to this paper. He expects to be able to report later upon more of such teeth with porcelain restorations made in this manner.

The essayist explained that in restorations by means of jacket crowns it is necessary to extend the porcelain at least one mm. under the gingivus and that the formation of the shoulder necessarily leaves many short enamel rods, some of which are more or less damaged in the preparation of the shoulder. He believes that these rods have not sufficient force to maintain their strength, and that they are easily broken down after the crown has been in position from two to five years. Formation of the shoulder partly in the dentine and partly in the enamel is not a good idea, the technic necessary for the preparation of the shoulder often lacerates the gum tissue, though in probably ninety per cent. of all cases it is possible for the skilful operator to remove enamel for the shoulder preparation without lacerating the gum tissue. That tissue heals readily if no permanent cause of irritation is left.

Cases in which restorations can be successfully made with filling materials should be excluded from this form of restoration. One must be certain that there is sufficient dentine about the pulp to insure its vitality and that the core of dentine is sufficiently strong to support the crown. It is not good practice to build up the core of the tooth with cement to carry the crown, and more often than not such practice results in failure. Radiographs must be studied for position of the pulp and pathological conditions.

When centrals, laterals, cuspids and bicuspids are so badly broken down that any other form of restoration will be unsightly, this form of service is indicated. In practice incisal cavities in anterior of an otherwise perfect tooth this form of restoration is practicable.

Impressions of labial and lingual surfaces of the teeth may be made in duplicate and provide replicas for study. These impressions are easily taken by heating a stick of modeling compound and laying it along the labial surfaces with the long axis of the stick at right angles to the long axis of the teeth, and then doing the same on the lingual surfaces. If the tooth to be crowned is out of alignment, it is well to construct the crown upon the original alignment.

The essayist sometimes uses infiltrative anesthesia, preparatory to preparation. The chair assistant should use upon the tooth large quantities of water to facilitate preparation and to avoid the infliction of pain. He then described four methods of removing enamel as practiced by four skilful dentists of his acquaintance and said that in the hands of a skilful operator all are probably equally good, but advised workers to adopt one technic and stick to it.

The first step in the preparation of a vital tooth to receive the crown is to cut back the incisal edge sufficiently to permit the thickness of porcelain suitable to the work the crown will be called upon to do and to incline this edge at right angles to the line of the force to which it will be exposed. A Jo-dandy disc is then passed up each side of the crown in such way as to remove enough enamel so that the widest part of the preparation will be at the cemento-enamel junction.

A cut is then made in the labial and lingual enamel going around the tooth parallel to the gingivus and close to it, but not touching it. During this work, plenty of water should be used upon the tooth either hot or of room temperature. With the finest knife-edged stone make cuts through the enamel across the labial and lingual surfaces at right angles to the long axis of the tooth. Then make similar cuts parallel to the long axis of the tooth until the labial and lingual surfaces present a series of square blocks which offer square corners to the enamel cleavers. The enamel can then be easily removed with the ordinary Case cleaver. This leaves a collar of enamel under the gum line which can be easily removed with planes similar to those used by the periodontists, only a little heavier.

It is very important to determine how far under the gum line the crown must extend. At least one mm. is advised in all cases and sometimes more is necessary.

It is very important that except for the enamel no living tissue should be removed from the tooth. In removing the enamel, the plane is put up under the free margin of the gum and drawn downward in line with the long axis of the tooth. The success of the crown depends upon the ability of the operator to plane the surface left after the enamel is removed as smooth as if it had been polished.

To get an impression for the crown, take a band of thirty-six gauge copper of a size which fits the prepared tooth so tightly that much force will be required to get it up in place. These bands, a half inch long, are made now by several manufacturers. Heat the band to a dull red heat and plunge it into alcohol which will render it very soft. Force the band upward as far as the gingivus and burnish it to an exact fit. Shape the cervical end of the band to perfectly fit the festoon of the gum. Determine how far up under the free margin of the gum the crown is to go and scratch upon the band a mark at this distance from its gingival margin and parallel to the crest of the gum. Also scratch a cross-mark upon the labial surface of the band to distinguish it from the lingual surface. Cut the band off to present a square edge on the festoon end.

The impression is taken by filling the band with modeling compound and heating it clear through. This is quite different from the technic followed in taking impressions for a denture and is very important because if the band is heated clear through, the excess will be forced out of the open end of the band. Otherwise it might be forced up under the gum and cause irritation. It is important also to be sure that the compound is seared to the sides of the band to prevent it from sliding out of position, because it will be very thin at the gingival margin of the band. Dip the impression into water heated to about 170° F. before putting it onto the tooth.

Do not force the impression under the gum at the present time or you may force the excess out in such way that you can not get the band well into position. Remove the impression and cut away the excess. Heat it all the way through again, place it upon the tooth and force it up until the line on the band which is parallel to the festoon of the gum comes at the edge of the festoon. If the impression is not perfect, repeat the process until it is.

When the impression is complete and satisfactory, wrap wax of about twenty-eight gauge around the copper band and make an amalgam model. Let this set three and a half or four hours. This gives a model with a shoulder, but it is not the shoulder of the finished case. The shoulder marks the exact point to which the cervical edge of the porcelain is to extend when finished.

To make the matrix for the crown, bend a piece of platinum foil around the amalgam die so that it encloses the die, and cut down to be a little longer than the model of the tooth. The two ends of the strip of platinum should meet on the lingual of the model and lap a little. By means of a wooden stick, outline the shoulder of the model in the matrix. Turn both edges of the strip, formed by the ends which met on the lingual, to form a tinner's joint. The essayist prefers to bake a little porcelain over this joint, at the cervical margin, though the joint may be sealed by fusing a little pure gold short of this point. This gold should seal only a very short portion of the joint and is intended only to hold the band in position. When this has been done, open the portion of the tinner's joint not sealed by the gold or porcelain and lap the porcelain ends, burnishing both to the model.

Make a shoulder crown in the usual way. Be careful not to accentuate the shoulder on the lingual, because the shoulder is to be ground away.

We have found that we can not grind this shoulder away after having given the crown the final glaze without chipping it. After the next to the last baking, which should be at 2400° or 2450° F., put the crown on the amalgam model and hold it with the forefinger on the amalgam and the thumb on the porcelain. With the cutting portion of an ordinary small disk stone revolving from the amalgam toward the porcelain, we first grind into the amalgam and then onto the edge of the crown until it has been thinned down to a sufficient degree.

The crown is then placed in the mouth and examination is made to find whether the proper anatomical form has been ground away or whether the porcelain is thick enough to induce a tension in the tissue which blanches the gum. If necessary, the operator grinds the crown again, until the blanching has disappeared and only the normal form of tension exists in the tissues.

The crown should then be boiled in a fifteen per cent. solution of sulphuric acid, followed by a saturated solution of bicarbonate of soda, which in turn is followed by clear boiling water. It is then held under the tap for some time to cleanse and neutralize it.

In order to support the crown during the last baking, mix ordinary fire-clay and fibre asbestos with a little water to a paste, out of which is formed a pedestal. Fit the crown to this pedestal. Remove the crown and put the pedestal in the furnace. It will become hard in about two minutes and will support the crown during the last baking, which should be at about 2560° F. for ten seconds.

It is very important to prevent seeping of fluids from the gingival tissue while setting the crown. This may be done by wiping the surface of the soft gingival tissues with trichloracetic acid on cotton. This will turn the gum white but will keep it dry for quite a long time. In a few hours the condition will pass away.

The best manner of cementing the crown is to paint a thin mixture of cement upon the tooth, but put little or no cement inside the crown. Before the crown is placed over the tooth upon which the cement has been painted, the crown should be wiped out with cotton which has been saturated with alcohol and then squeezed between the fingers. The cement should be one which sets well in the moisture of the mouth.

The outside of the crown should be oiled so that the excess cement shall come away in one piece. The crown should be teased to place as it can not be forced up over the stump.—*Dental Digest.*

